# Incidence and risk factors for redisplacement after closed reduction and instant rigid cast immobilization for paediatric distal radius fractures: a case control study

**DOI:** 10.1186/s13018-020-01672-z

**Published:** 2020-04-09

**Authors:** Lingde Kong, Jian Lu, Yanqing Zhou, Dehu Tian, Bing Zhang

**Affiliations:** Department of Orthopedics, The Third Hospital of Hebei Medical University, 139 Ziqiang Road, Shijiazhuang, Hebei 050051 People’s Republic of China

**Keywords:** Risk factors, Redisplacement, Cast immobilization, Paediatric, Distal radius fractures

## Abstract

**Abstract:**

**Background:**

The aim of this study is to record the incidence of redisplacement after closed reduction and instant rigid cast immobilization and to identify possible risk factors that may be associated with the redisplacement.

**Methods:**

We retrospectively reviewed paediatric patients who underwent closed reduction and instant rigid cast immobilization for simple distal radius fractures from 2014 to 2018. Patients were followed up at 1 week, 2 weeks, 3 weeks, and 6 weeks after casting. Redisplacement was diagnosed on the basis of image findings. Risk factors for redisplacement were evaluated in three aspects, which included patient-related, fracture-related, and cast-related factors.

**Results:**

A total of 123 children were included in this study. During follow-up, 31 patients (25.2%) showed redisplacement after closed reduction and cast immobilization. Twenty-two redisplacements happened within 1 week after treatment, 8 redisplacements happened between 1 and 2 weeks, and only one redisplacement happened after 2 weeks. In the multivariate analysis, associated ulna fracture (OR, 4.278; 95% CI, 1.773–10.320), initial translation ≥ 50% (OR, 9.148; 95% CI, 3.587–23.332), and 3-point index ≥ 0.40 (OR, 1.280; 95% CI, 1.159–1.401) were three independent factors that correlated with the incidence of redisplacement during follow-up.

**Conclusions:**

About a quarter of paediatric patients would develop redisplacement after reduction and immobilization with instant rigid cast. Patients with associated ulna fracture, severe initial translation, and high 3-point index have a higher risk to develop redisplacement.

## Background

Fractures of the distal radius are among the most common injuries presenting to orthopaedic surgeons, which involve up to 27% of all fractures in children [1]. In the majority of patients, the mechanism of injury is a direct fall and fractures are located in the metaphyso-diaphyseal area. Fractures in the paediatric population are different from those in adults, due to the ability of bone remodelling while the epiphysis remains open [2–4].

Both conservative and surgical treatments have been used for distal radius fractures [5–9]. Although several authors have advocated the use of operative methods of fixation such as percutaneous pinning in cases of severe angulation or displacement, some reports have shown similar cost and complication rates between closed reduction and percutaneous pinning [10]. In the clinical practice, closed reduction and cast immobilization is still the most common treatment of distal radius fractures in children.

Although closed reduction and cast immobilization has been accepted extensively, we have to confess that there is a very high rate of redisplacement following this treatment. The rates of redisplacement in cast have been reported to range from 21 to 39% after acceptable initial reduction [10, 11]. Factors that have been implicated in loss of reduction in children are numerous, and different studies undertaken to define their roles have not been able to give convincing results [12–15].

It is well known that application of a well-moulded cast or appropriate interosseous mould is critical in minimizing the risk of subsequent redisplacement [7, 16]. For traditional cast, skin irritation during casting is still a common complaint [17]. With the development of new synthetic materials in recent years, a newly instant rigid cast has been used in our hospital, which could potentially reduce patients’ suffering from itchiness, eruption, skin irritation, and bad odour. However, no studies have reported the incidence of redisplacement after this new type of cast and related risk factors.

In the current study, we tried to fill some of these gaps in clinical research by analysing paediatric patients who suffered from distal radius fractures. The aim is to record the incidence of redisplacement after closed reduction and instant rigid cast immobilization and to identify possible risk factors, which include patient-related factors, fracture-related factors, and cast-related factors that may be associated with the redisplacement.

## Materials and methods

### Patient population

We retrospectively reviewed paediatric patients who underwent closed reduction and instant rigid cast immobilization for simple distal radius fractures in our hospital between February 2014 and March 2018. The inclusion criteria were patients under 16 years old with distal fourth radius fractures confirmed by X-ray images. Patients with open fractures, epiphyseal injuries, associated dislocations, or fractures treated initially by K-wires or plate fixation were excluded from this study. Patients with unacceptable reduction (translation > 5 mm or angulation > 20°) were also excluded. The ethics committee of the Third Hospital of Hebei Medical University approved this research and waived the informed consent because this was a retrospective observational study, and all data were collected and analysed anonymously.

### Treatment and follow-up

Most patients underwent closed reduction under local block in the emergency room, and in some patients, reduction failed and these patients required additional manipulations under brachial block or general anaesthesia in the operating room. All reduction procedures were performed by experienced surgeons. Before and after treatment, anteroposterior (AP) as well as lateral X-ray tests were performed, and all distal forearm fractures were placed in short-arm casts. The instant rigid cast was used for all patients.

**Fig. 1.**
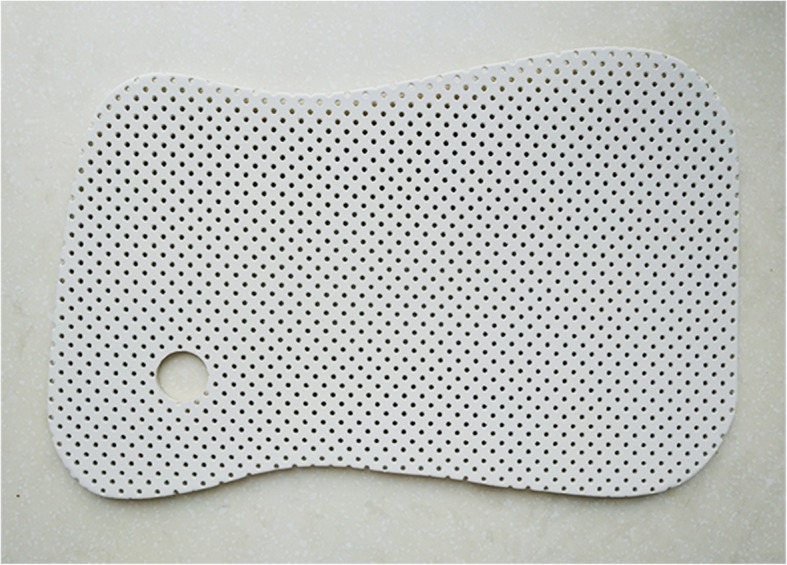
The appearance of the instant rigid cast

The instant rigid cast is made of thermoplastic material, which becomes soft when the ambient temperature exceeds 70° and turn to rigid soon after being exposed at room temperature (Fig. 1). In brief, spandex liner is put on the affected forearm first and then the reduction procedure is performed. The instant rigid cast is soaked in a 70° water bath for 3 to 5 min. After it softens, the performer takes it out of the water, covers it on the forearm of the patient, and modifies its shape to fit the limb with the assistance of elastic bandage. In the shaping process, the material should cover more than 5/6 of forearm circumference. A gap was left in order to observe the skin condition and swelling degree of the forearm. If the first shaping is unsuccessful, the cast can be put into the water for shaping again. After the shaping meets the requirement, the cast was fixed with thread gluing or ordinary bandage.

Patients were followed up at 1 week, 2 weeks, 3 weeks, and 6 weeks after casting. At each routine follow-up time, an X-ray test was performed. Redisplacement was defined by any modification from the initial postreduction radiograph. After fracture healing, the cast was removed and range of motion exercise was started.

### Parameter evaluation

**Fig. 2:**
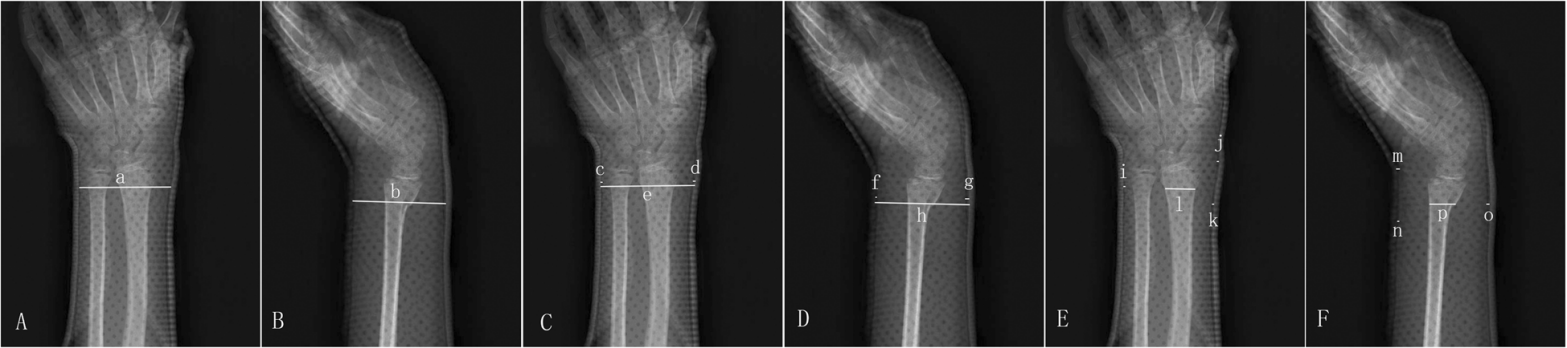
The measurement of cast related indices. **a**, **b** Cast index. Cast index = b/a. **c**, **d** Gap index. Gap index = (c + d)/e + (f + g)/h. **e**, **f** Three-point index. Three-point index = (i + j + k)/l + (m + n + o)/p

Risk factors for redisplacement were evaluated in three aspects, which included patient-related, fracture-related, and cast-related factors. Patient-related data, such as age and gender, were collected from electronic records. Fracture-related factors included distance from epiphysis, associated ulna fracture, translation, angulation, and adequacy of reduction. The adequacy of reduction was classified as anatomic, good, or fair. Anatomic reduction is complete anatomic fracture reduction with no translation or angulation, good reduction is < 10° of dorsal angulation or < 2 mm of translation, and fair reduction is less than a good reduction, with translation of between 2 and 5 mm or angulation of between 10 and 20° or any radial deviation of < 5° or a combination of 5 to 10° of dorsal angulation and < 2 mm of translation. The cast-related factors were cast index, padding index, and gap index. The indices were calculated as described by Bae et al. [18], and a brief description is listed below:

1. Cast index was estimated by measuring the inside diameter of the plaster in the lateral view as a ratio to the diameter in the AP view at the fracture site;
2. Gap index: [(radial fracture-site gap + ulnar fracture site gap)/inner diameter of cast in AP plane] + [(dorsal fracture site gap + volar fracture site gap)/inner diameter of cast in lateral plane];
3. Three-point index: [(distal radial gap + ulnar fracture site gap + proximal radial gap)/contact between fracture fragments in AP plane] + [(distal dorsal gap + volar fracture site gap + proximal dorsal gap)/contact between fracture fragments in lateral plane] (Fig. 2).

The radiographs taken after cast application were used for measurement of the various radiographic indices. Two blinded observers assess the radiological findings independently. For continuous variables, the mean values were used. For categorical variables, disagreements between the two observers were settled by discussion, and the third observer made the final decision in the case of no consensus could be reached.

### Data analysis

All statistical analysis was performed with the Statistical Package for Social Sciences software (version 17.0; SPSS Inc., Chicago, IL, USA). Categorical data were analysed for significance by the chi-square test; numerical data were analysed by independent *t* test or Mann-Whitney test. After univariate analysis, variables that might be potentially associated with redisplacement (*P* < 0.10) were entered into the multiple logistic regression analysis. Probability value < 0.05 was considered statistically significant.

## Results

After excluding 137 patients due to initial wire or plate fixation and 37 patients with unacceptable reduction, a total of 123 children were analysed in this study. Among these patients, 93 were male, and 30 were female. The mean age at the time of fracture was 10.3 ± 3.4 years. One hundred and twelve patients underwent closed reduction under local block in the emergency room, and 11 patients failed initial casting and required additional manipulations in the operating room. Seventy-nine patients underwent closed reduction one time, 32 patients underwent reduction twice, and 12 patients underwent reduction three or more times. X-ray images after treatment showed anatomical reduction in 45 patients, good reduction in 38 patients, and fair reduction in 40 patients (Table 1).

**Table 1:** Demographic data of paediatric patients with distal radius fractures

Variables	Values
Number of patients	123
Age (year)	10.3 ± 3.4
Gender	
Male	93 (75.6%)
Female	30 (24.4%)
Times of reduction	
One time	79 (64.2%)
Twice	32 (26.0%)
Three or more times	12 (9.8%)
Adequacy of reduction	
Anatomic	45 (36.6%)
Good	38 (30.9%)
Fair	40 (32.5%)
Number of patients with redisplacement	31
The time of redisplacement	
Within 1 week	22 (71.0%)
1–2 weeks	8 (25.8%)
2–3 weeks	1 (3.2%)
Further treatment	
Accept the redisplacement	24 (77.4%)
Remanipulation	4 (12.9%)
Surgical treatment	3 (9.7%)

During follow-up, 31 patients showed redisplacement after closed reduction and cast immobilization, with an incidence of 25.2%. Overall, 22 redisplacements happened within 1 week after treatment, 8 redisplacements happened between 1 and 2 weeks, and only one redisplacement happened between 2 and 3 weeks. After discovery of displacement, 4 patients underwent remanipulation, and 3 patients underwent surgical treatment with K-wires. For the other 24 patients, the amount of displacement was accepted with expectation of spontaneous remodelling with growth (Table 1). Other complications are extremely rare and without long-term sequelae. Only three patients (2.4%) had mild pressure sores after immobilization. Serious complications such as compartment syndrome, permanent median, or ulnar nerve dysfunction were not found.

In the univariate analyses of patient-related, fracture-related, and cast-related factors, we found that associated ulna fracture (*P* = 0.002), initial translation ≥ 50% contact (*P* < 0.001), initial angulation ≥ 20° (*P* = 0.033), non-anatomical reduction (*P* = 0.061), and high 3-point index (*P* < 0.001) were potential risk factors associated with redisplacement after closed reduction, while age, gender, distance from epiphysis, cast index, and gap index were not (*P* ≥ 0.10). The details of univariate analyses are listed in Table 2.

**Table 2:** The comparison of data in children with and without redisplacement

Risk factors	Redisplaced group (*n* = 31)	Undisplaced group (*n* = 92)	*P* value
Patient-related			
Age (year)	9.6 ± 3.6	10.5 ± 3.3	0.202
Gender			
Male	23	70	0.832
Female	8	22	
Fracture-related			
Distance from epiphysis (mm)	22.5 ± 8.8	20.4 ± 7.4	0.195
Associated ulna fracture			
Yes	14	16	0.002
No	17	76	
Initial translation			
< 50% contact	7	71	< 0.001
≥ 50% contact	24	21	
Initial angulation			
< 20°	15	64	0.033
≥ 20°	16	28	
Anatomical reduction			
Yes	7	38	0.061
No	24	54	
Cast-related			
Cast index	0.82 ± 0.10	0.79 ± 0.09	0.121
Gap index	0.11 ± 0.27	0.10 ± 0.26	0.835
Three-point index	0.58 ± 0.20	0.31 ± 0.17	< 0.001

In the further multivariate logistic regression, associated ulna fracture (OR, 4.278; 95% CI, 1.773–10.320), initial translation ≥ 50% (OR, 9.148; 95% CI, 3.587–23.332), and 3-point index ≥ 0.40 (OR, 1.280; 95% CI, 1.159–1.401) were three factors that correlated with the incidence of redisplacement during follow-up (Table 3).

**Table 3:** Multivariate logistic regression analysis of predictive factors associated with redisplacement after closed reduction and cast immobilization

	*P* value	Odds ratio	95% CI
Associated ulna fracture	0.001	4.278	1.773–10.320
Initial translation ≥ 50%	< 0.001	9.148	3.587–23.332
Initial angulation ≥ 20°	0.059	2.204	0.963–5.047
Non-anatomical reduction	0.224	1.720	0.714–4.145
Three-point index ≥ 0.40	< 0.001	1.280	1.159–1.401

*CI* confidence interval

## Discussion

Identification of the predictive factors for redisplacement may help surgeons to identify patients at the greatest risk for redisplacement and adjust their monitoring and follow-up decision. In this study, we reviewed patients who underwent closed reduction and cast immobilization. Our investigation revealed that the incidence of redisplacement was 25.2%. This high rate of redisplacement demonstrates the importance of being able to identify risk factors to prevent this poor outcome. The cause of redisplacement after reduction is likely to be multi-factorial. Patients with associated ulna fracture, initial translation ≥ 50%, and 3-point index ≥ 0.40 have a higher risk to develop redisplacement.

Some published series have reported that the loss of reduction rates varied from 21 to 39% of patients [10, 11]. We assumed that the criteria for conservative treatment are different in every study, and thus, the populations of these studies are heterogeneous. The inclusion of various patients with different risk factors may lead to the difference in redisplacement rate.

Whether concomitant distal ulna fracture is associated with higher risk of redisplacement is debated controversially in previous studies. For example, Mcquinn et al. reported that fractures of both distal forearm bone were not a risk factor for redisplacement [14]. However, Jordan et al. found that distal ulna fracture associated with radius fracture showed higher chance to became redisplaced [7]. In our study, we demonstrated that associated ulna fracture was an independent factor that correlated with the incidence of redisplacement.

The current study showed that initial translation is significantly correlated with redisplacement in instant rigid cast. The role of initial translation in redisplacement of fractures has been investigated by previous studies. Zamzam et al. attributed initial complete displacement as the single most important predictor of redisplacement [19]. Mani et al. reported a significant risk of redisplacement of distal radius fractures with higher degree of translation at the radial or ulnar fracture site. Their study also showed that the risk of failure was 60% when the translation was more than half of diameter of radius, whereas for less translation, the risk of redisplacement was 8% [20]. Initial translation was considered to be associated with soft tissue injury causing damage to the periosteum. High rate of redisplacement in severely displaced fractures is probably due to the loss of stability given by the periosteum and surrounding soft tissue structures. However, initial angulation was not found to be significantly associated with redisplacement in cast after multivariate analysis. We assumed that angulation deformity may lead to less damage to the periosteum and soft tissue than the translation deformity does.

We analysed three cast indices in our study, and statistical analysis indicated that cast quality using the 3-point index seems to be more reliable than other indices for redisplacement, which was consistent with several previous studies [16, 21]. To obtain a low 3-point index, we proposed the application of a well-moulded cast with 3-point bending to minimize the risk of subsequent displacement. Thus, further training to improve the quality of casting technique should be emphasized. Junior trainees should be trained to measure the 3-point index before accepting the reduction after casting to prevent late displacement.

In this study, we applied a new type of cast in paediatric distal radius fractures. The newly instant rigid cast can reduce patients’ suffering from itchiness, eruption, skin irritation, and bad odour. Also, the operation of the cast is much simpler and can be moulded repeatedly. Besides, the cast indices of the new cast were much lower than previous data of the traditional cast, which means that the new cast fits the forearm more tightly. We think this type of cast may be an eligible substitution for traditional cast in the treatment of distal radius fractures in children.

There are several limitations that need to be considered in this study. First of all, in order to obtain a homogeneous group, only children with simple distal radius fractures were included in the study. The results were not applicable to other populations with special fractures, such as comminuted fractures or concomitant epiphyseal injuries. Besides, only a limited number of predictive factors were investigated in our study; the involvement of other factors in further study may provide more information to us. Thirdly, this is a retrospective study. The study design and potential for bias are the typical restrictions of our study. Finally, this study only reported the clinical effect of instant rigid cast; further comparative studies with other external fixation method are necessary.

## Conclusion

In summary, we found that about a quarter of paediatric patients developed redisplacement after manipulation. The cause of redisplacement is likely to be multi-factorial. Patients with associated ulna fracture, fractures with severe initial translation, and high 3-point index of the cast have a higher risk to develop redisplacement. Therefore, deliberate treatment plan and close follow-up are necessary for cases with these risk factors.

## Data Availability

Not applicable.

## References

[1] Brudvik C, Hove LM (2003). Childhood fractures in Bergen, Norway: identifying high-risk groups and activities. J Pediatr Orthop.

[2] Rodriguez-Merchan EC. Pediatric fractures of the forearm. Clin Orthop Relat Res. 2005:65–72. 15738805

[3] Hove LM, Brudvik C (2008). Displaced paediatric fractures of the distal radius. Arch Orthop Trauma Surg.

[4] van der Sluijs JA, Bron JL (2016). Malunion of the distal radius in children: accurate prediction of the expected remodeling. J Child Orthop.

[5] Jordan RW, Westacott DJ (2012). Displaced paediatric distal radius fractures--when should we use percutaneous wires?. Injury.

[6] Ramoutar DN, Shivji FS, Rodrigues JN, Hunter JB (2015). The outcomes of displaced paediatric distal radius fractures treated with percutaneous Kirschner wire fixation: a review of 248 cases. Eur J Orthop Surg Traumatol.

[7] Jordan RW, Westacott D, Srinivas K, Shyamalan G (2015). Predicting redisplacement after manipulation of paediatric distal radius fractures: the importance of cast moulding. Eur J Orthop Surg Traumatol.

[8] Kamiloski M, Todorovik L, Memeti S (2018). The kapandji technique of closed reduction using sommer - pins in the treatment of completely dislocated fractures of the distal radius in children. Open Access Maced J Med Sci.

[9] Colaris JW, Allema JH, Biter LU (2013). Re-displacement of stable distal both-bone forearm fractures in children: a randomised controlled multicentre trial. Injury.

[10] Miller BS, Taylor B, Widmann RF (2005). Cast immobilization versus percutaneous pin fixation of displaced distal radius fractures in children: a prospective, randomized study. J Pediatr Orthop.

[11] Mclauchlan GJ, Cowan B, Annan IH, Robb JE (2002). Management of completely displaced metaphyseal fractures of the distal radius in children. A prospective, randomised controlled trial. J Bone Joint Surg Br.

[12] Arora R, Mishra P, Aggarwal AN, Anshuman R, Sreenivasan R (2018). Factors responsible for redisplacement of pediatric forearm fractures treated by closed reduction and cast: role of casting indices and three point index. Indian J Orthop.

[13] Asadollahi S, Ooi KS, Hau RC (2015). Distal radial fractures in children: risk factors for redisplacement following closed reduction. J Pediatr Orthop.

[14] Mcquinn AG, Jaarsma RL (2012). Risk factors for redisplacement of pediatric distal forearm and distal radius fractures. J Pediatr Orthop.

[15] Auer RT, Mazzone P, Robinson L, Nyland J, Chan G (2016). Childhood obesity increases the risk of failure in the treatment of distal forearm fractures. J Pediatr Orthop.

[16] Devalia KL, Asaad SS, Kakkar R (2011). Risk of redisplacement after first successful reduction in paediatric distal radius fractures: sensitivity assessment of casting indices. J Pediatr Orthop B.

[17] Derksen RJ, Commandeur JP, Deij R, Breederveld RS (2013). Swim cast versus traditional cast in pediatric distal radius fractures: a prospective randomized controlled trial. J Child Orthop.

[18] Bae DS (2008). Pediatric distal radius and forearm fractures. J Hand Surg Am.

[19] Zamzam MM, Khoshhal KI (2005). Displaced fracture of the distal radius in children: factors responsible for redisplacement after closed reduction. J Bone Joint Surg Br.

[20] Mani GV, Hui PW, Cheng JC (1993). Translation of the radius as a predictor of outcome in distal radial fractures of children. J Bone Joint Surg Br.

[21] Marcheix PS, Peyrou P, Longis B, Moulies D, Fourcade L (2011). Dorsal distal radius fractures in children: role of plaster in redisplacement of these fractures. J Pediatr Orthop B.

